# The Effects of Nut Consumption on Vascular Function

**DOI:** 10.3390/nu11010116

**Published:** 2019-01-08

**Authors:** Samantha Morgillo, Alison M. Hill, Alison M. Coates

**Affiliations:** 1 School of Health Sciences, University of South Australia, Adelaide 5001, Australia; morsp008@mymail.unisa.edu.au; 2 School of Pharmacy and Medical Sciences, University of South Australia, Adelaide 5001, Australia; alison.hill@unisa.edu.au

**Keywords:** tree nuts, peanuts, arterial stiffness, vascular reactivity, cardiovascular disease risk

## Abstract

Vascular stiffness can be measured using numerous techniques including assessments of central haemodynamics, aortic arterial stiffness, and indices of aortic wave reflection and endothelial dilatation. Impaired vascular function is associated with increased risk of cardiovascular disease (CVD). Epidemiological studies indicate that regular nut consumption reduces CVD risk, with one of the proposed mechanisms being via improvements in vascular function. This narrative review summarizes the evidence from a systematic search of the literature of the effects of tree nut and peanut consumption on measures of vascular function excluding flow mediated dilatation. A total of 16 studies were identified, with a mix of acute controlled studies (*n* = 3), an uncontrolled pre/post chronic study (*n* = 1), chronic crossover (*n* = 7) and parallel studies (*n* = 5). Nut types tested included almonds, peanuts, pine nuts, pistachios and walnuts, with dose and length of supplementation varying greatly across studies. Most studies (*n* = 13) included individuals at risk for CVD, according to various criteria. Findings were inconsistent, with ten studies reporting no significant changes in vascular function and six studies (one acute and five chronic studies) reporting improvements in at least one measure of vascular function. In summary, nuts have the potential to improve vascular function and future studies should consider the population, dose and length of nut supplementation as well as suitability of the different vascular function techniques.

## 1. Introduction

The nutrient profile of both tree nuts and peanuts has been proposed to be beneficial for cardiovascular health [1]. Nuts are rich in monounsaturated and polyunsaturated fats, folate, vitamin E, magnesium, potassium and arginine, as well as polyphenols and fibres in the skins [2]. Prospective epidemiological studies have consistently reported cardiovascular benefits associated with regular nut consumption [3]. This benefit has been linked with several proposed mechanisms, including altered cholesterol metabolism leading to improved lipid profiles, reduced oxidative stress, enhanced beta oxidation and reduced vascular inflammation, which promotes vascular health [4].

Nuts have positive effects on vascular endothelial function, with regular nut consumption associated with improved vasodilation (or reactivity) measured by flow mediated dilation (FMD) without affecting endothelium-independent vasodilatation [5,6]. While FMD is considered a prognostic marker for cardiovascular disease (CVD) [7], a number of other markers of vascular function are associated with increased risk of CVD, including elevated central haemodynamics, aortic arterial stiffness, and indices of aortic wave reflection [8,9], which may be improved with nut consumption.

Vascular reactivity is also measured using the device EndoPAT, which detects plethysmographic pressure changes in the finger tips caused by the arterial pulse, which are translated to a peripheral arterial tone (PAT) [10]. EndoPAT is easy to use but may be a less sensitive measure of vascular reactivity in comparison to FMD [11]. Comparatively, FMD uses ultrasound to measure dilation of the brachial artery in response to hyperemia but is dependent on the training of the operator, which limits its utility. Therefore, other assessments of vascular function, such as stiffness, are commonly used to evaluate risk. Vascular stiffness is an independent predictor for future cardiovascular events and mortality [12]. It can be assessed via pulse wave velocity (PWV), pulse wave analysis (PWA) and by assessing the inverse of arterial stiffness, which is arterial compliance. PWV examines the speed of the pulse wave generated by the heart along the arterial tree [13], where an increased velocity demonstrates stiffer arteries. PWA uses applanation tonometry to measure the pressure waveform from the brachial or radial artery to give the augmentation index (AI). Arterial compliance also involves analysis of the pulse wave contour from the radial artery to determine the elasticity index of the large (LAEI) and small artery (SAEI). This technique is limited by variation in stroke volume and blood pressure, which can modify the reflected waveform and alter the calculation [14]. The measure of SAEI estimates arterial function of the distal part of arterial circulation and a lower SAEI is considered to be predictive of cardiovascular disease events [15]. Another outcome that is indicative of stiffness is total peripheral resistance (TPR) [16], which can be assessed with impedance cardiography.

Vascular stiffness pathology is complex and the techniques used to assess this vary in their accuracy, precision, sensitivity and specificity. Along the arterial tree there are differences in muscularity and elasticity, hence local vascular measures are considered to be more quantitative and sensitive than systemic indices [17]. Whilst the various methods of assessing vascular stiffness described above are valid [18] and highly reproducible [19,20], it is important to note that the outcomes from different techniques may not be directly comparable [21].

Currently there has been no systematic evaluation of the literature related to the effects of nuts on markers of vascular function other than FMD [5,21]. Therefore, in order to better elucidate the mechanism(s) by which nuts might lower CVD risk, the aim of this narrative review containing a systematic search is to evaluate the evidence for the effect of nut consumption on vascular function (stiffness and reactivity) as assessed by all noninvasive functional measures other than FMD.

## 2. Materials and Methods

### 2.1. Eligibility Criteria

Only peer-reviewed intervention studies published in English evaluating at least one type of nut (tree nuts and/or peanuts) and their effects on vascular function measured as either vascular stiffness or vascular reactivity (other than FMD) were included.

#### 2.1.1. Types of Studies

All acute (immediate effects of a single dose of nuts, i.e., within hours) and chronic (nuts eaten for an extended period, i.e., weeks) intervention studies were considered.

#### 2.1.2. Types of Participants

Adult participants aged ≥18 years were considered.

#### 2.1.3. Types of Intervention

All types of tree nuts and peanuts were considered. Soy nuts were not included.

#### 2.1.4. Comparators

Both controlled and uncontrolled studies were considered.

#### 2.1.5. Types of Outcome Measures

Only noninvasive techniques used to assess vascular function were included, such as pulse wave velocity (PWV), pulse wave analysis (PWA), digital volume pulse (DVP), impedance cardiography and peripheral arterial tone via plethysmography (e.g., EndoPAT). The outcome measures derived from these techniques are listed in Table 1. Studies reporting the outcome measure of FMD were excluded, as this has recently been reviewed [21].

### 2.2. Information Sources

In August 2017, the following databases were systematically searched for relevant articles: Medline, CINAHL, Scopus, Cochrane and Embase. The reference lists of relevant review articles found in the search were also manually checked for studies missed by the database searches. This search was updated in July 2018 with no additional relevant papers identified.

### 2.3. Search

Search terms were collated into two key groups: nuts and vascular function. The following terms for nuts were searched in all databases: ‘nut or nuts or almond* or pistachio* or hazelnut* or walnut* or cashew* or macadamia* or pecan* or peanut* or “Corylus avellana” or “Prunus dulcis” or “Prunus amygdalus” or “Pistacia vera” or pistacia or juglans) or “Anacardium occidentale” or “Carya illinoinensis” or “Arachis hypogaea” or groundnut*’. The second group of terms were based on vascular function: “Arter* stiff*” or “Arter* complian*” or “arter* elasticit*” or “aortic stiffness*” or “vascular* stiff*” or “aortic complian*” or “aortic elasticit*” or “vascular* complian*” or “vascular* elasticit*” or “Arter* pressur*” or “aortic pressur*” or “vascular* pressur*” or laei OR saei or “pulse wave*” or “reflective index” or “Beta stiffness index” or “Augmentation Index” or “elasticity index” or “endopat” or “ankle brachial index” or “vascular resistance” or “systemic vascular resistance” or “peripheral resistance” or “arterial pressure wave” or “endothelial function”. The two groups were combined to search for relevant studies.

### 2.4. Study Selection

After removal of duplicates, studies were screened by title and abstract. The full text of all articles was then reviewed before final inclusion in the review.

### 2.5. Data Items

Extracted data from all studies included study design, sample size, participant characteristics (age, body mass index (BMI), sex and health status), the dietary intervention including the amount and type of nut, duration, placebo or control conditions, fed or fasted state for assessments and vascular function outcome.

## 3. Results

### 3.1. Study Selection and Study Designs

**Figure 1:**
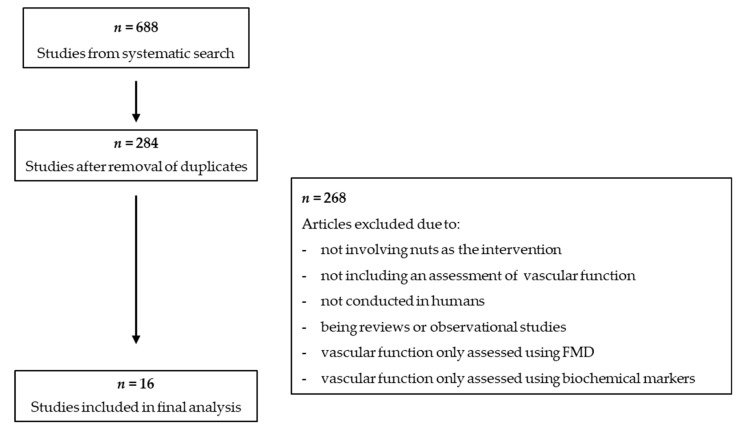
Flowchart of study selection process.

Figure 1 presents an overview of the literature search and study selection process. A total of 688 studies were identified in the initial search. After removing duplicates, 284 studies underwent a screening of the title and abstracts and 16 articles underwent a full text screening. Review articles were searched for relevant studies that may have been missed in the search, but none were identified. A total of 16 studies were included in this narrative review

### 3.2. Study Characteristics

The studies included in the review comprise three acute controlled studies, one uncontrolled pre/post chronic study, seven controlled chronic crossover studies and five controlled parallel studies. The majority of studies were conducted in middle-aged participants who were overweight or obese. Most studies included both male and female participants; Kasliwal et al. 2015 had a higher proportion of males compared to females. Three studies used only males [22,23,24] and one study used only females [25]. The population groups included in the studies varied greatly in health statuses, with only three studies focusing on healthy participants [22,23,26]. The remaining studies included population groups at risk for CVD, including overweight populations (*n* = 13) [23,24,25,27,28,29,30,31,32,33,34,35,36,37], participants with metabolic syndrome (*n* = 3) [28,30,37], elevated blood lipid levels (*n* = 4) [25,29], type 2 diabetes (*n* = 3) [31,33,36] or established coronary artery disease (*n* = 1) [34].

A range of outcome measures were reported in the studies (Table 2). The outcome measures for arterial stiffness were augmentation index (AI, *n* = 6), PWV (*n* = 3), small and large arterial elasticity (*n* = 1) and total peripheral resistance (TPR) (*n* = 2). Reactive Hyperemia Index (RHI), a measure of vascular reactivity, was reported in nine studies.

The most frequently evaluated nut type was walnuts, used in six out of the 16 studies [23,24,25,26,27,31]. The next most common nut types were pistachios (*n* = 4) [28,29,32,33] and almonds (*n* = 3) [22,34,36], with only one study conducted with peanuts alone [35], and two studies with mixed nuts [30,37].

### 3.3. Effect of Nuts on Vascular Function

#### 3.3.1. Acute Studies

Of the 16 studies, three evaluated the post-prandial effects of a single meal containing nuts on vascular function. The post-prandial duration ranged from 1–8 h. Two studies compared meals containing nuts against control meals; one study compared muffins containing nuts against muffins with sunflower oil [22]. The other study compared nuts to both white bread (50 g), or the combination of white bread (50 g) with butter and cheese [28]. These studies found no effect of nuts on vascular function assessed via AI [22,28] or RHI [28]. However, Berryman et al. [27] compared different subfractions of walnuts to baseline and found a treatment effect (*p* = 0.01) for RHI (vascular reactivity) but not for AI (vascular stiffness). They also reported that walnut oil favourably improved vascular reactivity compared to walnut skins.

#### 3.3.2. Pre/Post Non-Controlled Studies

Gulati et al. [36] used a three-week run in period and provided a standardised diet (60% carbohydrate, 15% protein, 25% fat) followed by 24 weeks of supplementation with almonds to provide 20% of total energy intake such that the almond diet contained 55% CHO, 17% PRO, and 28% fat. They found no significant improvement in PWV (*p* = 0.06) from 20% of total energy intake from almonds for 24 weeks.

#### 3.3.3. Chronic Crossover Studies

The majority of chronic studies included in this review used a cross-over design (8 out of 13). Three studies assessed vascular reactivity using EndoPAT (RHI) but no significant effects of nuts were reported [26,33,34]. These studies supplemented dietary intake with pistachios, almonds or walnuts for 4–8 weeks in doses ranging from 43 g/day to 128 g/day.

Vascular stiffness was assessed in seven studies. Barbour et al. [35] reported a 10% increase in small artery elasticity index (SAEI) after consumption of 56 g/day (females) or 84 g/day (males) of peanuts for 12 weeks (*p* = 0.008, effect size = 0.04). There was no improvement in AI after 15 g/day of walnuts [23] or 59–128 g/day of pistachios [33] for 4 weeks. Three studies included total peripheral resistance (TPR) as an outcome, with all studies reporting a significant improvement following nut consumption. West et al. 2012 [32] reported a significant reduction in TPR (−62.1 dynes x sec x cm^−5^, *p* < 0.0001) from 63–126 g/day of pistachios for 4 weeks. Sauder et al. [33] also reported significant benefits of pistachios (59–128 g/day for 4 weeks) on TPR (−3.7 ± 2.9%, *p* = 0.004). West et al. 2010 [24] reported a 4% reduction in TPR (*p* ≤ 0.05) from 37 g/day of walnuts for 6 weeks.

A range of control diets were administered, including habitual diets [23,35], moderate to high total fat (30–35% total energy) and saturated fat (10–15% total energy) diets [24,26], and cholesterol-lowering diets, such as the American Heart Association [33] and National Cholesterol Education Program Step 1 diet [34].

#### 3.3.4. Chronic Parallel Studies

Five chronic studies used a parallel study design. These studies ranged in duration from 4 to 12 weeks and included nut doses of 28 g to 40 g per day. The majority of studies (four out of five) assessed changes in vascular function using Endo-PAT with mixed results. Holt et al. [25] reported a significant increase in RHI (2.63 ± 0.10 vs. 2.23 ± 0.13, *p* = 0.025) with 40 g/day of walnuts compared to a control diet of 5 g/day of walnuts for 4 weeks and also a better post-prandial response in the 40 g/day group. However, Lopez-Uriarte et al. [30] and Djousse et al. [31] reported no change in RHI after 12 weeks’ supplementation with 30 g/day mixed nuts or 28 g/day walnuts, respectively. Lee et al. [37] reported no change in RHI after 6 weeks of 30 g/day of mixed nuts. These differences may in part be due to sample size and population characteristics. Djousse et al. [31] had a small sample of 26 participants (13 in each group), 15% of whom had coronary artery disease. Lopez-Uriarte et al. [30] and Lee et al. [37] used a low dose of 30 g/day of mixed nuts. However, the one parallel study that looked at vascular stiffness reported significant results. Kasliwal et al. [29] reported significant treatment effects for left brachial ankle pulse wave velocity (Ba-PWV), and carotid-femoral pulse wave velocity (Cf-PWV) (*p* = 0.01 and 0.037, respectively) from 40 g/day of pistachios for 4 weeks.

While the control diets differed among studies, the majority included non-nut groups. Djousse et al. [31] compared the nut intervention to a habitual diet, whereas Kasliwal et al. [29], Lee et al. [37] and Lopez-Uriarte et al. [30] used modified diets that focused on healthier eating patterns as their control diet.

## 4. Discussion

This review found that studies evaluating the effect of tree nut and peanut consumption on vascular function have shown mixed effects, with six out of 16 studies reporting a benefit associated with nut consumption and ten showing no effect. Acute crossover studies failed to demonstrate that nut consumption can affect vascular reactivity or vascular stiffness post-prandially. Although acute vascular reactivity was improved with consumption of walnut oil, this was not compared to a control group. Evidence from chronic studies suggest that regular consumption of nuts can improve TPR, with all three studies that assessed this outcome reporting a positive effect, with two of these studies assessing the effects of pistachios and one the effects of walnuts. Improved arterial stiffness was also observed following peanut and pistachio consumption compared to a nut-free diet and lifestyle modification, respectively. Only one study in walnuts observed improvements in vascular reactivity. Therefore, while there is evidence from some studies that nuts can improve arterial function, the findings are inconsistent.

When considering the effects of nuts on vascular function the population type is important. The studies in healthy populations did not find significant improvements, whereas six (out of the 12) studies in people with CVD risk factors reported significant changes in at least one measure of vascular function [24,25,29,32,33,35]. Comparatively there were no improvements in vascular stiffness or reactivity in people with established coronary artery disease [34], possibly due to the relatively short duration of the intervention and the impaired responsiveness to vasodilatory stimuli observed in individuals with coronary artery disease [38,39]. Population groups with CVD risk factors may be more likely to have improvements, as there is a level of vascular dysfunction that can be corrected. This is consistent with the results of Del Gobbo et al. 2015 [40], who found stronger effects from nut consumption in reducing lipoproteins for those who had type 2 diabetes compared to those who were healthy. Therefore, in patients who are healthy who exhibit good vascular function, or in patients with established disease who have very poor vascular function, nuts may not provide any benefit. However, in patients with risk factors for disease in whom vascular function is moderately impaired nuts may be beneficial.

A similarity between this review and that performed by both Xiao et al. [21] and Neale et al. [5] was that the majority nut type was walnuts. Xiao et al. [21] also included two studies using pistachios and one study each of almonds and hazelnuts. Xiao et al. found that the beneficial effect of improving vascular reactivity was limited to walnuts [21]. This may be due to the small sample of studies of other nuts compared to walnuts. Another reason may be due to the fatty acid profile of walnuts containing higher amounts of plant-derived omega-3 α-linoleic acid compared to other nut types [41]. This fatty acid has been associated with cardioprotective benefits, such as being antithrombotic, anti-atherogenic and anti-inflammatory [42]. The current review, however, failed to find consistent results from walnuts, with only two of the chronic studies showing an improvement in vascular function. The nutrient profile of nuts may be influenced by seasonal variability and climate conditions in different growing regions, which could contribute to different responses across studies [43,44]. There has been one study assessing changes in baroreflex sensitivity with consumption in walnuts and cashews compared with a control diet [45]. Whilst not a direct measure of vascular function (hence this study was not included in Table 2), baroreflex sensitivity is an important mechanism in the regulation of blood pressure and hence this study warrants highlighting [46]. Schutte et al. [45] found that walnuts and cashews both significantly altered baroreflex sensitivity in people with metabolic syndrome following 8 weeks of supplementation. Interestingly, these nuts had opposite effects, such that cashews exerted a positive effect (defined as an autonomic shift towards the parasympathetic side), while walnuts had a negative effect. The authors postulate that these differences may be related to the difference in fatty acid profile, but this finding warrants further investigation.

In comparing the findings in this review and with previous reviews focused on FMD outcomes, both the doses of nuts provided and length of supplementation should be considered. In the current review, the range of nut doses (15–128 g/day) is lower compared to the doses used in the studies analysed by Xiao et al. [21] (52–128 g/day) and Neale et al. [5] (18–85 g/day). This may have contributed to the observed inconsistencies between reviews. Xiao et al. [21] reported that nut consumption <67 g/day was more effective at improving FMD compared to >67 g/day. One possible explanation may be due to reduced participant compliance with higher doses; consequently, dose response studies should be undertaken to address this issue. At this stage, there is insufficient evidence to indicate an optimum dosage of nuts to improve vascular function.

Furthermore, inconsistency in results may be due to differences in study durations. The duration of the studies included by Xiao et al. [21] ranged from 4–24 weeks, with an average length of 13 ± 7 weeks. Neale and colleagues [5] included studies ranging from 4 weeks to 5 years, although the majority were less than 3 months in duration. Studies included in this review had a similar range, varying from 4–24 weeks, with an average of 9 ± 6 weeks. Previous dietary interventions with anthocyanins have been able to demonstrate changes in arterial function acutely [47], and interventions with fish oil have shown improvements after six weeks to one year [48], indicating that changes are possible within this relatively short timeframe. Other possible reasons for different effects of nut consumption on vascular function could be related to the background diets of participants, socioeconomic status, education levels or genetic differences [49,50].

## 5. Conclusions

This purpose of this review was to summarise the evidence for improved vascular stiffness or reactivity following consumption of tree nuts and peanuts. Based on the studies included in this review, there is some evidence for an improvement in vascular stiffness, with 5 of 13 chronic studies reporting significant reductions following nut consumption. Unfortunately, the small number of studies, along with the diverse range of outcome measures, nut types evaluated and duration of interventions limits our ability to draw a definitive conclusion. Similar to recommendations from other recent reviews in FMD, we emphasise the need for more nut intervention studies assessing functional measures of vascular health.

## Figures and Tables

**Table 1:** Techniques used to assess vascular function and outcome measure.

Technique	Outcome Measure					
	Reactive Hyperemia Index (RHI)	Augmentation Index (AI)	Pulse Wave Velocity (PWV)	Large and Small Artery Elasticity Index (LAEI and SAEI)	Total Peripheral Resistance (TPR)	Stiffness Index (SI)
Peripheral arterial tone (PAT)	✓	✓				
Pulse wave analysis		✓		✓		
Pulse wave velocity			✓			
Impedance cardiography					✓	
Digital volume pulse (DVP)						✓

AI—augmentation index, DVP—digital pulse wave, LAEI—large artery elasticity index, PAT—peripheral arterial tone, PWA—pulse wave analysis, PWV—pulse wave velocity, RHI—reactive hyperemia index, SAEI—small artery elasticity index, SI—stiffness index, TPR—total peripheral resistance.

**Table 2:** Study characteristics.

Authors	Country	Duration	Assessment Times	*N* M:F	Health status	Age (years)	BMI (kg/m^2^)	Nut Type/Form Eaten	Dose (day)	Control	Vascular Function Measure	Findings
	**Acute studies (cross-over)**											
Berry et al. [22]	UK	8 h	2, 4, 6, 8 h	20 20:0	Healthy	All: 26 ± 4	All: 25 ± 3	Muffins containing Almond kernel Almond oil/almond flour	54 g (fat)	Muffin Sunflower oil	AIx	No significant difference between groups
Berryman et al. [27]	USA	4 h	0, 4 h	15 6:9	OW/OB	All: 49 ± 8	All: 29 ± 4	Walnuts Whole walnuts Nut meat Nut oil Nut skin	85 g	No control	RHI AIx	Significant reduction in RHI with walnut skins compared with baseline and higher RHI with walnut oil compared with skins
Kendall et al. [28]	Canada	3 h	1, 3 h	20 8:12	MetS	All: 54 ± 8	All: 38 ± 8	Pistachios	85 g	White bread White bread +butter +cheese	AIx RHI	No significant change with pistachio containing meal for either AIx or RHI but AIX increased in the meal with cheese
	**Pre/post study**											
Gulati et al. [36]	India	24 wk	0, 24 wk	50 27:23	T2D	All: 46 ± 9	All: 28 ± 5	Almonds	20%E	pre/post design without a temporal control	PWV	No significant change in PWV (trend toward improvement in pulse wave velocity (*p* < 0.06))
**Cross-over studies**												
Barbour et al. [35]	AUS	12 wk	0, 12 wk	61 29:32	OW	All: 65 ± 7	All: 31 ± 4	Peanuts	F: 56 g M: 84 g	Habitual diet	SAEI LAEI	Increase in SAEI during peanut phase (10%, *p* = 0.008)
Chen et al. [34]	USA	6 wk	0, 6 wk	45 18:27	CAD	All: 62 ± 9	All: 30 ± 5	Almonds	85 g	Step 1 diet (NCEP)	Cf-PWV Cr-PWV RHI	No significant change
Din et al. [23]	UK	4 wk	0, 4 wk	30 30:0	Healthy	All: 23 ± 3	All: 25 ± 2	Walnuts	15 g	Habitual diet (no walnuts)	AIx	No significant change
Sauder et al. [33]	USA	4 wk	0, 4 wk	30 15:15	T2D	All: 56 ± 8	All: 31 ± 1	Pistachios	20%E 59–128 g	AHA diet	RHI AIx	No significant change
West et al. [24]	USA	6 wk	0, 6 wk	20 20:0	HC	All: 49 ± 2	All: 29 ± 1	Walnuts	LA diet: 37 g walnut; +15 g walnut oil +19 g flax ALA diet: 37 g walnut; +15 g walnut oil	Average American diet	TPR	Significantly lower TPR during the LA and ALA diets compared with control diet (*p* = 0.02)
West et al. [32]	USA	4 wk	0, 4 wk	28 10:18	HC	All: 48 ± 22	All: 21–35	Pistachios	32–63 g (10%E) 63–126 g (20%E)	Low-fat diet (25% fat, 8% SFA)	TPR	Significant reduction in TPR with 20%E dose (−62.1 dynesx sec x cm^−5^, *p* < 0.0001)
Wu et al. [26]	Germany *	8 wk	0, 8 wk (+4 h post-prandial)	40 10:30	Healthy	All: 60 ± 6	All: 25 ± 4	Walnuts	43 g	Western diet	RHI	No significant change
	**Chronic parallel studies**											
Djousse et al. [31]	USA	12 wk	0, 12 wk	26 10:16	T2D	All: 65 ± 12	C: 34 ± 9 I: 30 ± 5	Walnuts	28 g	Habitual diet	RHI	No significant change
Holt et al. [25]	USA	4 wk	0, 4 wk (+4 h post-prandial each visit)	38 0:38	HC	C: 60 ± 6 I: 60 ± 4	C: 24 ± 3 I: 25 ± 4	Walnuts	40 g	5 g Walnuts	RHI AIx	Increase in fasting RHI over time in 40 g vs. 5 g group (2.63 ± 0.10 vs. 2.23 ±0.13, *p* = 0.025), Reduction in post-prandial change in RHI 40 g vs. 5 g group after 4 weeks (acute on chronic) No significant changes in Aix
Kasliwal et al. [29]	India	12 wk	0, 12 wk	60 50:10	Dys	All: 39 ± 8	C: 26 ± 3 I: 28 ± 5	Pistachios (salted)	40 g	LSM	Ba-PWV (Right, Left + Av) Cf-PWV	Reduced left Ba-PWV and Cf-PWV with pistachios vs. LSM (*p* < 0.05 for 2-way ANOVA)
Lee et al. [37]	Korea	6 wk	0, 6 wk	60	MetS	All:35–65	All: 27 ± 2	Mixed nuts (walnuts, pine nuts, peanuts)	30 g (15 g, 7.5 g, 7.5 g)	Prudent diet	RHI	No significant change
Lopez-Uriarte et al. [30]	Spain	12 wk	0, 12 wk	50 28:22	MetS	All: 52 ± 8	C: 30.0 (28.6–31.4) I: 31.6 (30.5–32.8)	Mixed nuts (walnuts, almonds, hazelnuts)	30g (15 g, 7.5 g, 7.5 g)	AHA diet	RHI	No significant change

American Heart Association diet—limit saturated fats, salt and alcohol, choose wholegrains and fruits/vegetables, eat fish twice a week. Life style modification: 50–55% carbohydrate 15–18% proteinPRO, 25–30% Fat. Prudent diet—high intake of vegetables, fruits, legumes, wholegrains, fish and poultry. Western diet—50%CHO, 15% PRO, 35% fat (15% SFA). *—participants in this study were identified as Caucasian, %E—percentage of total energy, AHA—American Heart Association, AIx—augmentation index, ALA—alpha-linoleic diet, AUS—Australia, Ba-PWV—brachial ankle pulse wave velocity, C—control group, CAD—coronary artery disease, Cf-PWV—carotid-femoral pulse wave velocity, CI—confidence interval, Cr-PWV- carotid-radial pulse wave velocity, Dys—dyslipidemia, F—female, g—grams, h—hour, HC—hypercholesterolemia, I—intervention group, LA—linoleic diet, M—male, MetS—metabolic syndrome, NCEP—National Cholesterol Education Program, LAEI—large artery elasticity index, LSM—lifestyle modification, OB—obese, OW—overweight, PWV—pulse wave velocity, RHI—reactive hyperemia index, SAEI—small arterial elasticity index, SFA—saturated fat, T2D—type 2 diabetes, TPR—total peripheral resistance, UK—United Kingdom, USA—United States of America, wk—week.

## References

[1] Coates A.M., Hill A.M., Tan S.Y. (2018). Nuts and Cardiovascular Disease Prevention. Curr. Atheroscler. Rep..

[2] Barbour J.A., Howe P.R., Buckley J.D., Bryan J., Coates A.M. (2014). Nut consumption for vascular health and cognitive function. Nutr. Res. Rev..

[3] Kelly J.H., Sabate J. (2006). Nuts and coronary heart disease: An epidemiological perspective. Br. J. Nutr..

[4] Kim Y., Keogh J.B., Clifton P.M. (2017). Benefits of nut consumption on insulin resistance and cardiovascular risk factors: Multiple potential mechanisms of actions. Nutrients.

[5] Neale E.P., Tapsell L.C., Guan V., Batterham M.J. (2017). The effect of nut consumption on markers of inflammation and endothelial function: A systematic review and meta-analysis of randomised controlled trials. BMJ Open.

[6] Huang Y., Zheng S., Wang T., Yang X., Luo Q., Li H. (2018). Effect of oral nut supplementation on endothelium-dependent vasodilation—A meta-analysis. VASA Z. Gefasskrankh..

[7] Ras R.T., Streppel M.T., Draijer R., Zock P.L. (2013). Flow-mediated dilation and cardiovascular risk prediction: A systematic review with meta-analysis. Int. J. Cardiol..

[8] Laurent S., Cockcroft J., Van Bortel L., Boutouyrie P., Giannattasio C., Hayoz D., Pannier B., Vlachopoulos C., Wilkinson I., Struijker-Boudier H. (2006). Expert consensus document on arterial stiffness: Methodological issues and clinical applications. Eur. Heart J..

[9] Taylor J.L., Curry T.B., Matzek L.J., Joyner M.J., Casey D.P. (2014). Acute effects of a mixed meal on arterial stiffness and central hemodynamics in healthy adults. Am. J. Hypertens..

[10] Moerland M., Kales A.J., Schrier L., van Dongen M.G.J., Bradnock D., Burggraaf J. (2012). Evaluation of the EndoPAT as a Tool to Assess Endothelial Function. Int. J. Vasc. Med..

[11] Allan R.B., Delaney C.L., Miller M.D., Spark J.I. (2013). A comparison of flow-mediated dilatation and peripheral artery tonometry for measurement of endothelial function in healthy individuals and patients with peripheral arterial disease. Eur. J. Vasc. Endovasc. Surg..

[12] Vlachopoulos C., Aznaouridis K., Stefanadis C. (2010). Prediction of cardiovascular events and all-cause mortality with arterial stiffness: A systematic review and meta-analysis. J. Am. Coll. Cardiol..

[13] O’Rourke M.F., Gallagher D.E. (1996). Pulse wave analysis. J. Hypertens. Suppl..

[14] DeLoach S.S., Townsend R.R. (2008). Vascular stiffness: Its measurement and significance for epidemiologic and outcome studies. Clin. J. Am. Soc. Nephrol. CJASN.

[15] Hom E.K., Duprez D.A., Jacobs D.R., Bluemke D.A., Brumback L.C., Polak J.F., Peralta C.A., Greenland P., Magzamen S.L., Lima J.A. (2016). Comparing Arterial Function Parameters for the Prediction of Coronary Heart Disease Events: The Multi-Ethnic Study of Atherosclerosis (MESA). Am. J. Epidemiol..

[16] Shepherd J.R., Hart E.C., Curry T.B., Charkoudian N., Joyner M.J., Casey D.P. (2010). Relationship between total peripheral resistance and measures of central artery wave reflection. FASEB J..

[17] Stoner L., Young J.M., Fryer S. (2012). Assessments of arterial stiffness and endothelial function using pulse wave analysis. Int. J. Vasc. Med..

[18] Weber T., O’Rourke M.F., Lassnig E., Porodko M., Ammer M., Rammer M., Eber B. (2010). Pulse waveform characteristics predict cardiovascular events and mortality in patients undergoing coronary angiography. J. Hypertens..

[19] Wilkinson I.B., Fuchs S.A., Jansen I.M., Spratt J.C., Murray G.D., Cockcroft J.R., Webb D.J. (1998). Reproducibility of pulse wave velocity and augmentation index measured by pulse wave analysis. J. Hypertens..

[20] Papaioannou T.G., Stamatelopoulos K.S., Georgiopoulos G., Vlachopoulos C., Georgiou S., Lykka M., Lambrinoudaki I., Papamichael C.M., Stefanadis C.I. (2009). Arterial wave reflections during the menstrual cycle of healthy women: A reproducibility study. Hypertension.

[21] Xiao Y., Huang W., Peng C., Zhang J., Wong C., Kim J.H., Yeoh E.K., Su X. (2018). Effect of nut consumption on vascular endothelial function: A systematic review and meta-analysis of randomized controlled trials. Clin. Nutr..

[22] Berry S.E., Tydeman E.A., Lewis H.B., Phalora R., Rosborough J., Picout D.R., Ellis P.R. (2008). Manipulation of lipid bioaccessibility of almond seeds influences postprandial lipemia in healthy human subjects. Am. J. Clin. Nutr..

[23] Din J., Aftab S., Jubb A., Carnegy F., Lyall K., Sarma J., Newby D., Flapan A. (2011). Effect of moderate walnut consumption on lipid profile, arterial stiffness and platelet activation in humans. Eur. J. Clin. Nutr..

[24] West S., Krick A., Klein L., Zhao G., Wojtowicz T., McGuiness M., Bagshaw D., Wagner P., Ceballos R., Holub B. (2010). Effects of diets high in walnuts and flax oil on hemodynamic responses to stress and vascular endothelial function. J. Am. Coll. Nutr..

[25] Holt R., Yim S.J., Shearer G., Keen C., Djurica D., Newman J., Shindel A., Hackman R. (2014). Correlation of lipoprotein epoxide content to microvascular function after short-term walnut intake. FASEB J..

[26] Wu L., Piotrowski K., Rau T., Waldmann E., Broedl U., Demmelmair H., Koletzko B., Stark R., Nagel J., Mantzoros C. (2014). Walnut-enriched diet reduces fasting non-HDL-cholesterol and apolipoprotein B in healthy Caucasian subjects: A randomized controlled cross-over clinical trial. Metabolism.

[27] Berryman C., Grieger J., West S., Chen C., Blumberg J., Rothblat G., Sankaranarayanan S., Kris-Etherton P. (2013). Acute consumption of walnuts and walnut components differentially affect postprandial lipemia, endothelial function, oxidative stress, and cholesterol efflux in humans with mild hypercholesterolemia. J. Nutr..

[28] Kendall C., West S., Augustin L., Esfahani A., Vidgen E., Bashyam B., Sauder K., Campbell J., Chiavaroli L., Jenkins A. (2014). Acute effects of pistachio consumption on glucose and insulin, satiety hormones and endothelial function in the metabolic syndrome. Eur. J. Clin. Nutr..

[29] Kasliwal R., Bansal M., Mehrotra R., Yeptho K., Trehan N. (2015). Effect of pistachio nut consumption on endothelial function and arterial stiffness. Nutrition.

[30] Lopez-Uriarte P., Nogues R., Saez G., Bullo M., Romeu M., Masana L., Tormos C., Casas-Agustench P., Salas-Salvado J. (2010). Effect of nut consumption on oxidative stress and the endothelial function in metabolic syndrome. Clin. Nutr..

[31] Djousse L., Petrone A., Gaziano J. (2015). Effects of walnut intervention on endothelial function among people with type 2 diabetes: A randomized trial. FASEB J..

[32] West S., Gebauer S., Kay C., Bagshaw D., Savastano D., Diefenbach C., Kris-Etherton P. (2012). Diets containing pistachios reduce systolic blood pressure and peripheral vascular responses to stress in adults with dyslipidemia. Hypertens..

[33] Sauder K., McCrea C., Ulbrecht J., Kris-Etherton P., West S. (2015). Effects of pistachios on the lipid/lipoprotein profile, glycemic control, inflammation, and endothelial function in type 2 diabetes: A randomized trial. Metabolism.

[34] Chen C., Holbrook M., Duess M., Dohadwala M., Hamburg N., Asztalos B., Milbury P., Blumberg J., Vita J. (2015). Effect of almond consumption on vascular function in patients with coronary artery disease: A randomized, controlled, cross-over trial. Nutr. J..

[35] Barbour J.A., Howe P.R.C., Buckley J.D., Bryan J., Coates A.M. (2017). Cerebrovascular and cognitive benefits of high-oleic peanut consumption in healthy overweight middle-aged adults. Nutr. Neurosci..

[36] Gulati S., Misra A., Pandey R.M. (2017). Effect of Almond Supplementation on Glycemia and Cardiovascular Risk Factors in Asian Indians in North India with Type 2 Diabetes Mellitus: A 24-Week Study. Met. Syn. Relat. Disord..

[37] Lee Y.J., Nam G.E., Seo J.A., Yoon T., Seo I., Lee J.H., Im D., Bahn K.N., Jeong S.A., Kang T.S. (2014). Nut consumption has favorable effects on lipid profiles of Korean women with metabolic syndrome. Nutr. Res..

[38] Schindler T.H., Hornig B., Buser P.T., Olschewski M., Magosaki N., Pfisterer M., Nitzsche E.U., Solzbach U., Just H. (2003). Prognostic value of abnormal vasoreactivity of epicardial coronary arteries to sympathetic stimulation in patients with normal coronary angiograms. Arterioscler. Thromb. Vasc. Biol..

[39] Heitzer T., Schlinzig T., Krohn K., Meinertz T., Munzel T. (2001). Endothelial dysfunction, oxidative stress, and risk of cardiovascular events in patients with coronary artery disease. Circulation.

[40] Del Gobbo L.C., Falk M.C., Feldman R., Lewis K., Mozaffarian D. (2015). Effects of tree nuts on blood lipids, apolipoproteins, and blood pressure: Systematic review, meta-analysis, and dose-response of 61 controlled intervention trials. Am. J. Clin. Nutr..

[41] US Department of Agriculture, Agricultural Research Service, Nutrient Data Laboratory USDA National Nutrient Database for Standard Reference. https://www.ars.usda.gov/northeast-area/beltsville-md-bhnrc/beltsville-human-nutrition-research-center/nutrient-data-laboratory/.

[42] Sala-Vila A., Ros E. (2011). Mounting evidence that increased consumption of a. linolenic acid, the vegetablen. 3 fatty acid, may benefit cardiovascular health. Clin. Lipidol..

[43] Abdallah I.B., Tlili N., Martinez-Force E., Rubio A.G.P., Perez-Camino M.C., Albouchi A., Boukhchina S. (2015). Content of carotenoids, tocopherols, sterols, triterpenic and aliphatic alcohols, and volatile compounds in six walnuts (Juglans regia L.) varieties. Food Chem..

[44] Rabadán A., Pardo J.E., Pardo-Giménez A., Álvarez-Ortí M. (2018). Effect of genotype and crop year on the nutritional value of walnut virgin oil and defatted flour. Sci. Total. Environ..

[45] Schutte A.E., Van Rooyen J.M., Huisman H.W., Mukuddem-Petersen J., Oosthuizen W., Hanekom S.M., Jerling J.C. (2006). Modulation of baroreflex sensitivity by walnuts versus cashew nuts in subjects with metabolic syndrome. Am. J. Hypertens..

[46] Swenne C.A. (2013). Baroreflex sensitivity: Mechanisms and measurement. Neth. Heart J..

[47] Fairlie-Jones L., Davison K., Fromentin E., Hill A.M. (2017). The Effect of Anthocyanin-Rich Foods or Extracts on Vascular Function in Adults: A Systematic Review and Meta-Analysis of Randomised Controlled Trials. Nutrients.

[48] Pase M.P., Grima N.A., Sarris J. (2011). The effects of dietary and nutrient interventions on arterial stiffness: A systematic review. Am. J. Clin. Nutr..

[49] Morris A.A., Patel R.S., Binongo J.N.G., Poole J., Al Mheid I., Ahmed Y., Stoyanova N., Vaccarino V., Din-Dzietham R., Gibbons G.H. (2013). Racial differences in arterial stiffness and microcirculatory function between Black and White Americans. JAMA.

[50] Trudel X., Shipley M.J., McEniery C.M., Wilkinson I.B., Brunner E.J. (2016). Socioeconomic status, education, and aortic stiffness progression over 5 years: The Whitehall II prospective cohort study. J. Hypertens..

